# Single Session of Calcium Hydroxylapatite, Incobotulinumtoxin A, and Hyaluronic Acid for Gluteal Aesthetic Improvement

**DOI:** 10.1111/jocd.70227

**Published:** 2025-05-12

**Authors:** David Di Sessa, Carla de Sanctis Pecora, Rossana Cantanhede Farias de Vasconcelos, Gustavo Limongi Moreira

**Affiliations:** ^1^ DSL Clinica, Private Practice São Paulo Brazil; ^2^ Clinica Dermatologie, Private Practice São Paulo Brazil; ^3^ Department of Dermatology Universidade de Santo Amaro São Paulo Brazil; ^4^ DSL Clinica Medica, Private Practice São Paulo Brazil

**Keywords:** aging, buttocks, calcium hydroxylapatite, hyaluronic acid, incobotulinum toxin A, sagging

## Abstract

**Background:**

While various techniques exist to treat the buttocks, few combine multiple products in a single session, offering both aesthetic and logistical benefits.

**Aims:**

To evaluate the aesthetic improvement of the buttocks in patients treated with a combination of calcium hydroxylapatite‐carboxymethylcellulose (CaHA‐CMC), Incobotulinumtoxin A (IncoBoNT‐A), and cohesive poly‐densified matrix hyaluronic acid (CPM‐HA V) in one session.

**Patients/Methods:**

This retrospective observational study involved women aged from 35 to 55 years who underwent the combined technique. Data were collected from the patient's charts, and photos were taken before treatment, and at 30 and 90 days post‐procedure. Two independent physicians evaluated cellulite (5‐point scale) and flaccidity (4‐point scale) in all patients. Aesthetic improvements were assessed by patients and physicians using the GAIS (5‐point) scale.

**Results:**

Ten women aged 45.3 (±5.2) years participated. Initially, 80% presented *moderate* to *severe* flaccidity, and 30% and 90% presented *moderate* to *very severe* cellulite in relaxed and contracted buttocks, respectively. After 90 days, flaccidity improved in 9/10 patients, and cellulite in 4/10 and 9/10 patients in static and dynamic assessments, respectively. There was a moderate correlation between the assessments at baseline and after 90 days. The aesthetic appearance of the buttocks was rated as *much improved* or *very much improved* by 100% of the patients and 60% of the physicians after 90 days.

**Conclusion:**

A single‐session combined application of CaHA‐CMC, IncoBoNT‐A, and CPM‐HA effectively improved flaccidity and cellulite, resulting in high patient satisfaction.

## 1 Introduction

The demand for body aesthetic procedures has been increasing in recent years [1]. Minimally invasive procedures, initially designed for the facial area, have been lately applied across multiple anatomical areas such as arms [2], thighs [3], neck/decolletage [4], and buttocks [5]. The buttocks have a particularly important appeal, especially for women [6]. The aging process is characterized by a loss of muscle mass, fat, and supporting connective tissue, in addition to changes in the bone structure in the gluteal region. As a result, the contour and surface of the area of the buttocks deform, leading to flaccidity, cellulite, volume loss, and decreased aesthetic satisfaction [6].

Attempts to minimize these processes using injectable products and energy‐based devices have emerged as useful tools to improve the aesthetic conditions of the gluteal region. Combined techniques have been proposed recently, leading to long‐lasting results [7, 8]. Calcium hydroxylapatite‐carboxymethylcellulose (CaHA‐CMC) is one of the most used biostimulators in the buttocks. It provides excellent tissue biocompatibility and a long‐term increase in collagen and elastin production [9]. Another important cosmetic product used for aesthetics purposes but not very usual for buttocks is cohesive poly‐densified matrix hyaluronic acid (CPM‐HA). Despite being not so common, it has significant water‐retaining capacity in the skin and increases skin elasticity, maintaining the volume of the dermis and improving skin quality [10]. IncobotulinumtoxinA (IncoBoNT‐A) is widely used for aesthetic and therapeutic purposes, mainly in the face, and has been investigated as a method to reduce cellulite by relaxing the fascia muscle [11] and also blocking the myofibroblasts [12, 13] located in the septal adipocytes.

In clinical practice, it is observed that treating buttocks is difficult, especially in cases where cellulite is significant. For these cases, the combination of two or more products with or without energy‐based devices is common. However, these procedures are typically performed over several sessions and may take a long time to achieve satisfactory results, which can increase costs and reduce patient adherence and satisfaction. Thus, the combination of three safe and effective products that are widely used in aesthetic treatments in only one session could be interesting for the patients. Therefore, the objective of this study was to evaluate the aesthetic improvement of the buttocks after a single session of combined treatment with CaHA‐CMC, IncoBoNT‐A, and CPM‐HA.

## 2 Materials and Methods

This was a retrospective observational study of women between 35 and 55 years old who had complaints about aesthetic changes in their buttocks due to flaccidity, cellulite, and/or loss of contour. These patients underwent the combined application of CaHA‐CMC, CPM‐HA and IncoBoNT‐A at a private clinic in São Paulo from April 2023 to January 2024. The criterion for inclusion was if they had never undergone any cosmetic procedure in the area and/or had not applied permanent products. After the Institutional Review Board Committee approved the study (number 6.941.771), patients were invited to participate and signed the informed consent form.

Before treatment, patient assessments were conducted, and dorsal, oblique, and right and left lateral plane photographs were taken in a standard upright position with the buttocks both relaxed and contracted. Each patient was treated with a combination of six syringes of hyperdiluted (1:2) CaHA‐CMC (Radiesse; manufactured by Merz North America Inc., Franksville, WI, USA), a 100 U vial of IncoBoNT‐A reconstituted in 2 mL of saline solution (Xeomin; manufactured by Merz Pharmaceuticals GmbH, Frankfurt, Germany), and six syringes of high G' and high cohesivity CPM‐HAV (BeloteroVolume; manufactured by Anteis S.A., Lonay, Switzerland).

**FIGURE 1:**
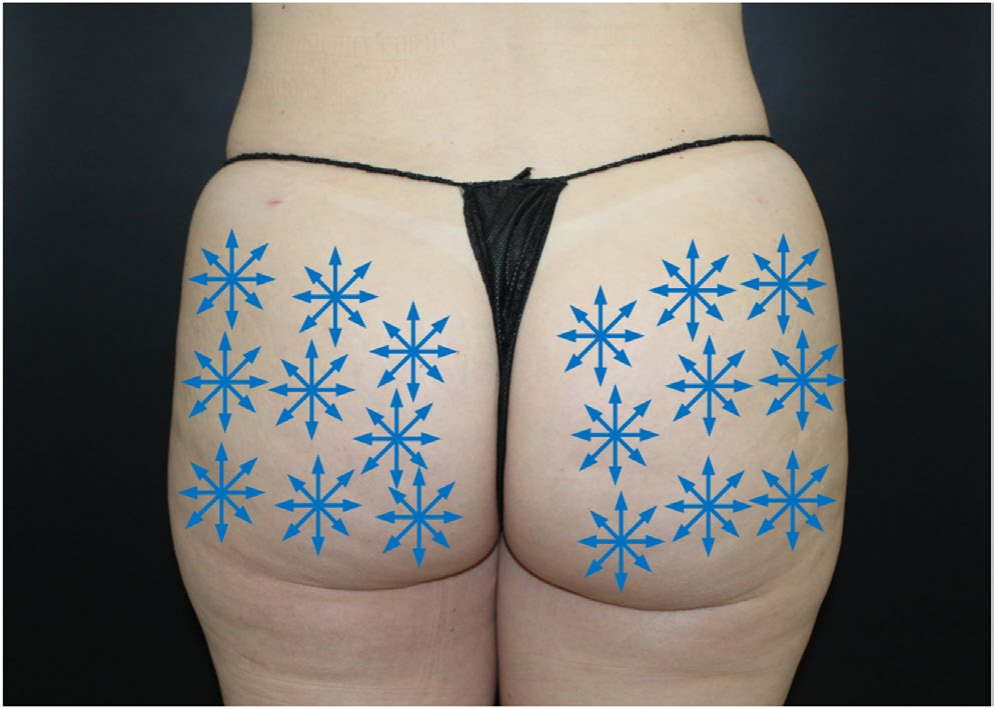
Scheme of boundaries to carry out the procedure.

After preparing each product separately, using a 20 cc LuerLock syringe and a female‐to‐female transfer adapter, the 6 CPM‐HAV syringes, 6 CaHA‐CMC syringes, and 100 U of IncoBoNT‐A were added to 10 mL of saline solution and were mixed directly in two syringes, totaling at least 20 transfers between the syringes to ensure a homogeneous mixture. In total, a mixture of 27 mL was obtained, maintaining the CaHA‐CMC dilution rate of 1:2. For the application, nine marks were made on each gluteal band to define the treatment areas and facilitate the visualization of the insertion sites, as shown in Figure 1. The markings should cover the entire gluteal area affected by cellulite. Adjacent areas should only receive markings once the entire cellulite‐affected area had been fully covered. Additionally, nine anesthetic buttons (2% lidocaine + vasoconstrictor) were applied at cannula entry points. Using a 22x70 G cannula, 1.5 mL of the mixture was applied in the subdermal plane at each mark using the *fanning* technique immediately after reconstitution. No massage was performed after the application, and a gentle bandage covered the entry site for 12 h.

After the procedure, patients were followed for 90 days, with assessments and photos repeated on day 30 and day 90. Patients and physicians applied the Global Aesthetic Improvement Scale (GAIS) on days 30 and 90. The degree of gluteal flaccidity and cellulite was analyzed by two independent physicians, in accordance with each other, using the *Skin Laxity Posterior Thighs and Buttocks* [3] and the *Scale for Cellulite Dimples on the Buttocks* [14] before and after 30 and 90 days for all patients. Adverse effects were collected at the procedure and during all the follow‐up.

### 2.1 Statistical Analysis

Age was expressed as means and standard deviation, while ordinal variables from the scales were presented as median and interquartile range (25%–75%) or as absolute and relative numbers. To compare the improvement in flaccidity and cellulite and find possible differences across the three assessments (pretreatment, 30 days‐post, and 90 days‐post), the categorical grades of the scales were transformed into rank scores: *no cellulite/no flaccidity* (0), *mild* (1), *moderate* (2), *severe* (3); and for the cellulite scale, *very severe* (4) [3, 14]. To compare the scores of the scales with an asymmetrical distribution, Friedman's test was applied, with Conover's post hoc correction for multiple comparisons.

Similarly, the aesthetic satisfaction levels of the GAIS scale were transformed into rank scores as follows: *worse* (1), *unchanged* (2), *improved* (3), *much improved* (4), and *very much improved* (5). The GAIS scores at 30‐ and 90‐days posttreatment were compared using the Wilcoxon test for patients and physicians decision scores, separately. The agreement between patients and physicians aesthetic satisfaction scores was tested using the Kappa test. Additionally, these ranks were used to analyze the possible correlation of the cellulite scores during relaxed and contracted assessments at 90 days post‐procedure, using the Spearman test.

The level of significance established was 5%. Good correlation was considered if *r* ≥ 0.80, moderate if r was between 0.80 and 0.50, and weak if 0.50 < *r* ≥ 0.30 or no correlation if *r* < 0.30 [15]. The JASP program was used to carry out analysis.

## 3 Results

During the study period, 12 patients were eligible, but two of them did not return for follow‐up. The remaining patients were females with an average (±SD) age of 45.3 (±5.2) years. Among them, 60% complained about both cellulite and flaccidity, 20% about flaccidity alone, 10% about contour, and 10% about volume loss. In the initial assessment, 80% had *moderate* (2) or *severe* (3) flaccidity, and 30% and 90% had *moderate* (2) to *very severe* (4) cellulite in a relaxed and contracted position of the buttocks, respectively. No adverse effects were observed in the sample.

**FIGURE 2:**
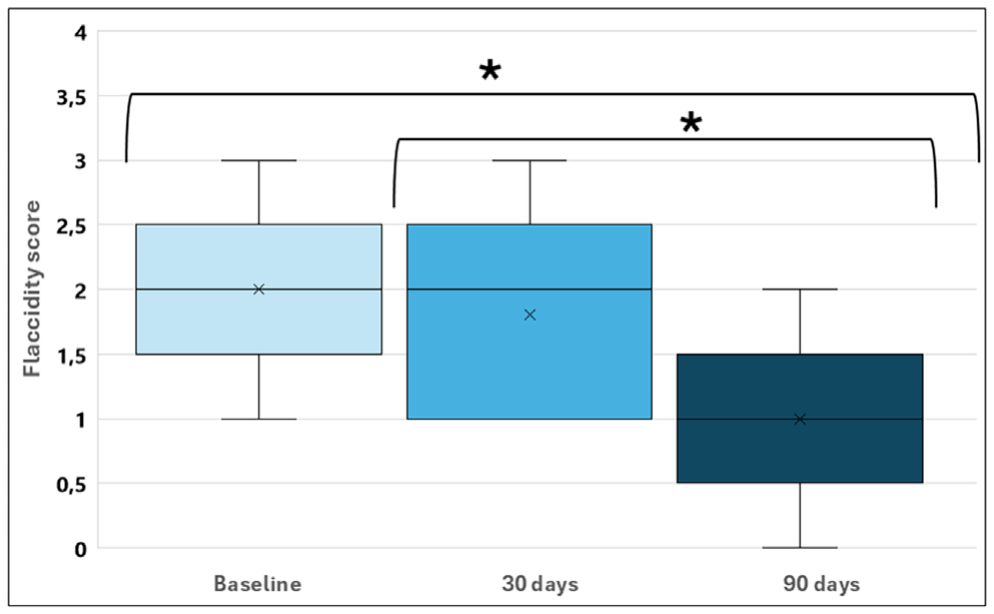
Physician‐evaluated flaccidity scores at baseline, 30 days posttreatment, and 90 days posttreatment. Within subjects' effects: *p* < 0.001. Post hoc comparisons: *p < 0.001. Boxplot: X: Represents the means. The boxes represent the 25th, median, and 75th percentiles. The straight lines represent the minimum (above the box) and maximum (below the box).

After 90 days, flaccidity improved by at least 1 point in 9/10 patients compared to baseline, and in 7/10 patients compared to day 30 posttreatment. The decrease in flaccidity occurred throughout the assessment, as shown in Figure 2.

**FIGURE 3:**
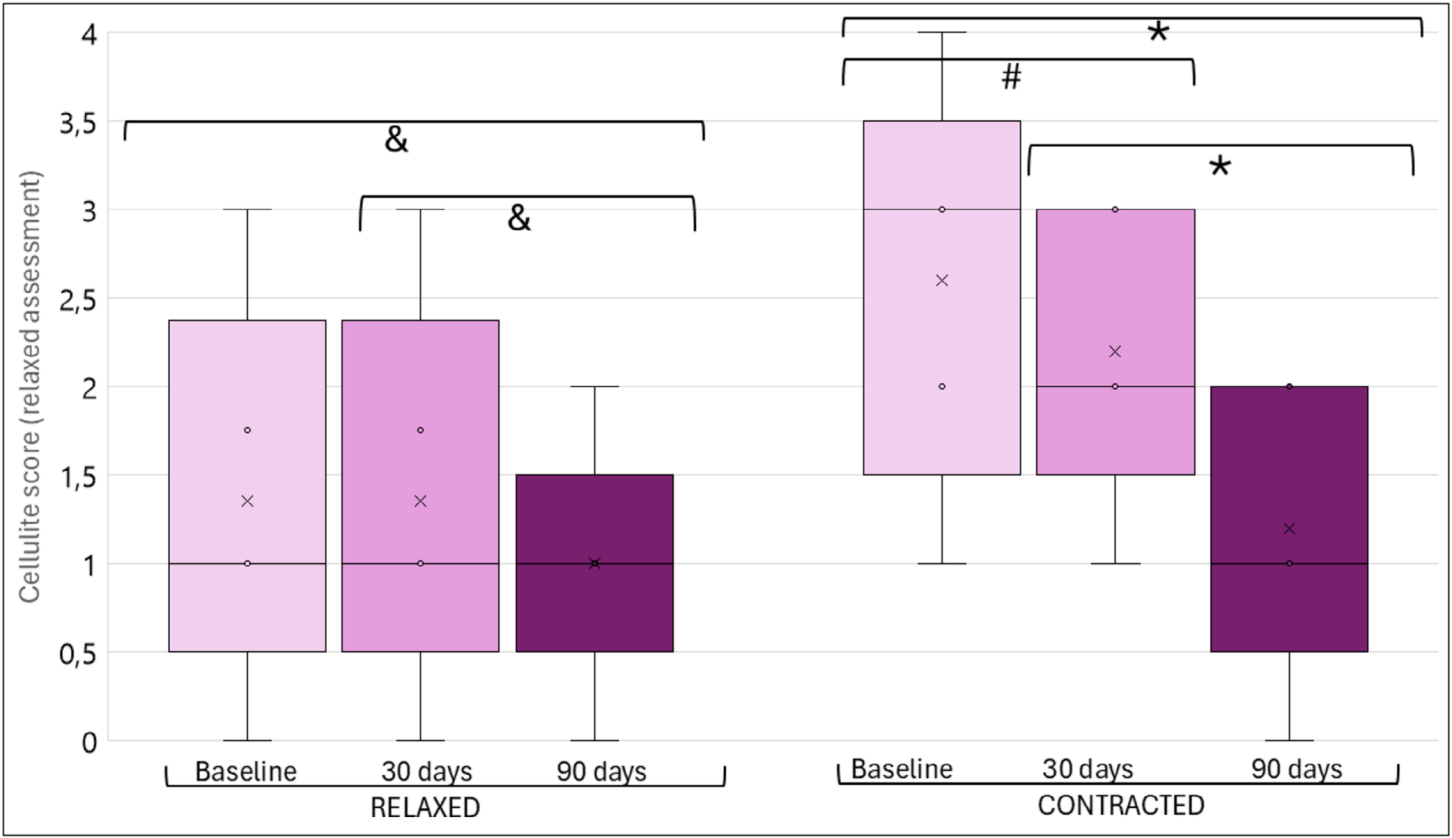
Cellulite scores of the sample with the buttocks relaxed and contracted at baseline, 30, and 90 days. Within subjects' effects: Relaxed (*p* = 0.041) and contracted (*p* < 0.001). Post hoc comparisons: *p < 0.001; # *p* = 0.009; & *p* = 0.021. Boxplot: X: Represents the means. The boxes represent the 25th, median, and 75th percentiles. The straight lines represent the minimum (above the box) and maximum (below the box).

**FIGURE 4:**
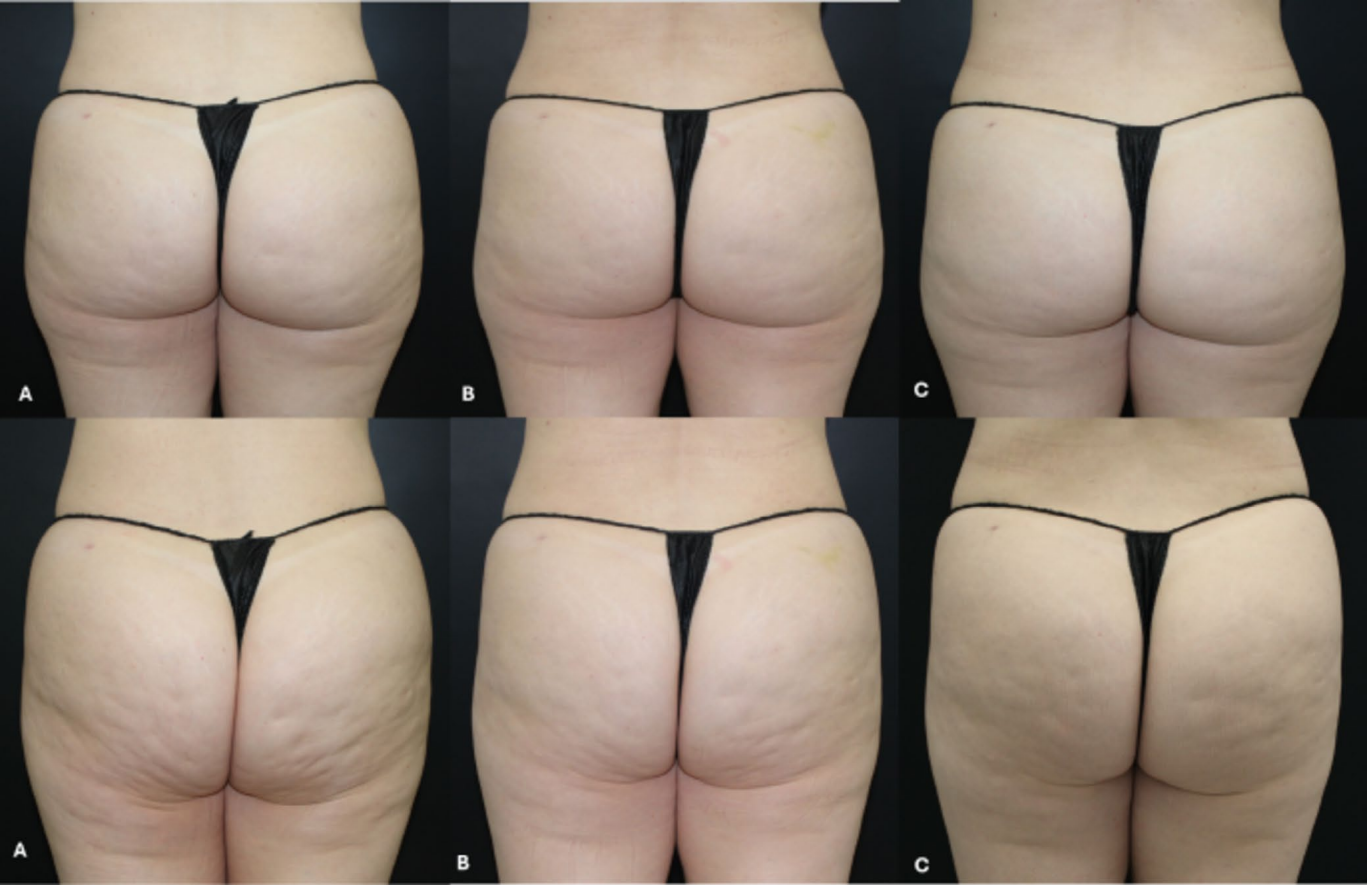
Example of a patient assessment with buttocks relaxed (static assessment) on the top and contracted (dynamic assessment) on the bottom of the figure. (A) Pre‐procedure, (B) 30 days post‐procedure, and (C) 90 days post‐procedure.

Cellulite also showed an improvement, as shown in Figure 3. After 90 days, cellulite scores improved by at least 1 point in 4/10 patients when the buttocks were relaxed compared with the baseline, and after 30 days, only 10% improved within 30 days compared to baseline. This improvement was significant when comparing the 90‐day assessment with the baseline and with 30 days after the procedure, and comparing the 90‐day assessment with 30 days after the procedure (Figures 3 and 4).

When the buttocks were contracted, after 90 days, cellulite scores improved by at least 1 point in 10/10 patients (8 patients with 1 point, 1 patient with 2 points, and 1 patient with 3 points) compared to baseline, and in 4/10 patients compared to day 30 posttreatment. Comparing day 90 to day 30, 8/10 patients improved by at least 1 point on the *Scale for Cellulite Dimples on the Buttocks*. The analysis confirmed that the improvement persisted throughout all assessments when the buttocks were contracted (Figures 3 and 4). Furthermore, there was a moderate correlation between the assessments when the buttocks were contracted and relaxed at baseline (*r* = 0.712; *p* = 0.021) and after 90 days (*r* = 0.646; *p* = 0.044) and a weak correlation after 30 days of the procedure (*r* = 0.477; *p* = 0.163).

**FIGURE 5:**
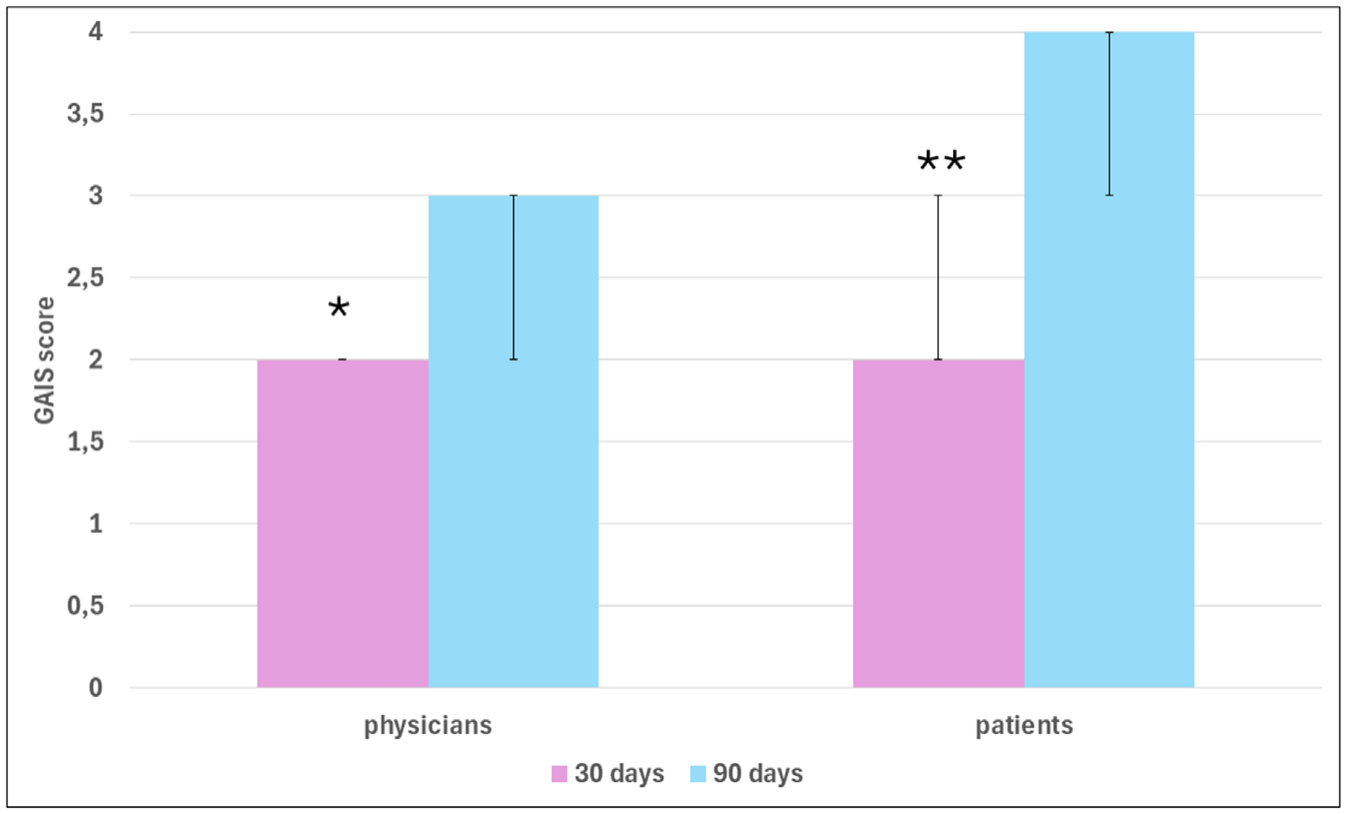
Patients' and physicians' scores of aesthetic satisfactions after 30 and 90 days post‐treatment. Data expressed in median and interquartile (25%–75%). Within groups differences between 30‐ and 90‐days post‐procedure. *p = 0.032 **p = 0.004.

After 90 days, all patients rated the appearance of their buttocks as *much improved* (4) or *very much improved* (5), while physicians gave these scores to 6/10 patients. Both groups noted improvements between day 30 and day 90 posttreatment (Figure 5). Despite the improvement cited by both patients and physicians, there was no agreement in their scoring (Kappa at 30 days = 0.167; Kappa at 90 days = 0.074).

## 4 Discussion

The buttocks are a key element of sexual attraction and an integral part of the concept of female beauty [6, 16]. Consequently, the changes that occur deriving from aging prompt women to seek ways to restore their appearance. In our study, we used a combination of three injectable cosmetics, in a single session, to decrease flaccidity and cellulite and to enhance buttocks contour as well as patient satisfaction. Our findings showed a significant reduction in flaccidity and cellulite among patients, accompanied by aesthetic satisfaction enhancement.

The rationale for injecting three different products is based on their specific actions. Hyperdiluted CaHA‐CMC, due to its biostimulating properties, stimulation of endogenous collagen types I and III, elastin production, increased glycosaminoglycan and angiogenesis, and regenerates extracellular matrix structure and function [17]. CPMV provides additional volume and creates a more lifted and contoured appearance [10, 18] and IncoBoNT‐A aims at relaxing the myofibroblasts present in the fibrous septa and fascia, as well as within the subcutaneous fat layer [12, 13], reducing the dimples and depressions.

Hybrid techniques using a combination of CaHA and HA for body contouring have been previously reported [5] and they have gained increasing attention due to their dual benefits of immediate volume enhancement and long‐term biostimulation effects. Their results showed an increase in buttock enhancement, demonstrating a significant volumetric improvement and contour refinement in buttock enhancement, with high patient satisfaction and minimal adverse effects. This reinforces the potential of hybrid solutions not only for facial applications but also for body treatments, addressing concerns such as sagging, cellulite, and skin texture. The ability of CaHA to stimulate collagen production while HA provides immediate structural support aligns with the evolving preference for minimally invasive procedures with natural‐looking outcomes.

Dimples are primarily caused by the shortening and tightening of fibrous septa within the subcutaneous fat layer [14, 19]. The more the septa shorten, the more the skin is pulled down. BoNT‐A can directly inhibit the differentiation of fibroblasts into myofibroblasts, suggesting its potential in treating conditions characterized by excessive fibrosis [12, 13] as occurs in cellulite. Moreover, it also has a regenerative role, improving blood flow perfusion [20]. The findings suggest that IncoBoNT‐A increases the expression of vascular endothelial growth factor (VEGF), cluster of differentiation 31 (CD‐31), and inducible nitric oxide synthase (INOS) [20]. In our study, cellulite assessment showed important improvements, mainly during dynamic assessment (in contraction) when the septal adipocytes are more evident, justifying the application of BoNT‐A combined with the other products.

During the assessment, when the buttocks were relaxed and contracted, on day 90, less than half of the patients showed improvement when the buttocks were relaxed, compared to baseline, and only 10% showed improvement at 30 days. On the other hand, when the buttocks were contracted, up to 90% showed improvement compared to baseline, with 20% by two points or more, and 40% showed improvement after 30 days. These better results during contraction and after several months probably occurred due to the initial relaxation and consequently strengthening of the fibrous septa caused by the botulinum toxin [7] in the first moment, followed by the action of CaHA‐CMC, regenerating the fibrous septa in the long term.

Supporting these findings, our analysis revealed a moderate correlation between the assessments of contracted and relaxed buttocks at baseline (*R* = 0.724, *p* = 0.004) and after 90 days (*R* = 0.646, *p* = 0.044), but a weak correlation after 30 days (*R* = 0.163, *p* = 0.163). The results suggest that there is initially some agreement between static and dynamic assessments, which diverge after the procedure, likely due to the greater action of botulinum toxin on contraction rather than relaxing. However, in the long term, they converge again, probably due to the significant increase in collagen production caused by CaHA‐CMC.

Flaccidity frequently occurs in areas where a large amount of adipose tissue is found, such as the buttocks. Since this area holds significant appeal and a constant concern [6, 21], mainly among women, improving its contour and density is generally desirable. Our results showed that between the first and the third months after the procedure, the improvement in flaccidity was particularly notable, probably due to the long‐term effects of CaHA‐CMC collagen stimulation. Studies demonstrated that the mechanism of action between the microspheres present in CaHA‐CMC and fibroblasts, throughout their mechanoreceptors, stimulates the production and the reorganization of collagen types I and III into a more organized and firmer network, as if in younger skin [22]. Furthermore, CaHA‐CMC stimulates the production of elastin, proteoglycans, angiogenesis, and neovascularization [23]. These changes can improve skin quality and roughness, resulting in a firmer, denser collagen network, increased dermal thickness, and overall improved skin appearance [5], presenting the best effects after several months, as corroborated by our results.

The improvement in flaccidity, cellulite, and consequently, the contour of the buttocks was reflected in the aesthetic satisfaction rated by patients and physicians. Applying a validating scale (GAIS) for both, all patients reported that their aesthetic appearance improved “very much” or “much” after 90 days, and 40% reported this at 30 days. In contrast, only 60% of the physicians rated the improvement at the same level after 90 days, and only 10% did so after 30 days.

Interestingly, patients reported higher scores than physicians on days 30 and 90, indicating a mismatch between their assessments, as shown by Kappa analysis. Several factors could explain this discrepancy. Satisfaction and expectations are personal and subjective matters, and even a small improvement can lead to greater personal satisfaction and a positive impact on their self‐esteem and confidence. Conversely, physicians focus on the technical and objective aspects of the procedures and their short‐ and medium‐term results, without considering the emotional aspects as patients do.

There are two potential points of concern: the risk of adverse events and the costs of the procedure. All three products are widely used worldwide for different purposes and have shown minimal adverse effects, most of which are related to technical errors. In our study, no adverse effects were observed except for mild pain during the injection, which demonstrates that the procedure is safe when performed with the proper technique. Regarding the cost, although it might initially seem more expensive than other isolated techniques, it may be similar or lower than other procedures, as the application is done in only one session. Moreover, the improvement of dimples achieved through BoNT‐A and the short‐term results obtained by HA application balance the cost‐effectiveness of the procedure.

The small sample size and the absence of a controlled group using another technique or a multi‐arm design using only one or two products could represent limitations to the results, potentially limiting the generalization of the findings. However, despite this limitation, the use of safe and efficient products, widely used in the aesthetics approach, in this procedure demonstrated significant benefits, enhancing patient satisfaction and improving the condition of the buttocks. These positive outcomes suggest that the procedure has a meaningful impact, which could be further validated with larger, more comprehensive studies. Additionally, the aesthetic benefits reported by patients underscore the importance of considering both physical and mental health improvements in evaluating the procedure's overall effectiveness.

In conclusion, the study highlights the potential benefits of a single‐session combined application of CaHA, CPM‐HA V, and IncoBoNT‐A in improving flaccidity and cellulite, with high levels of patient satisfaction.

## Author Contributions

All authors contributed to the work reported. Davi Di Sessa: Conception, study design, execution, acquisition of data, analysis and interpretation (70%); took part in drafting, revising a/o critically reviewing the article (30%). Carla de Sanctis Pecora: Conception, study design, execution, acquisition of data, analysis and interpretation (10%); took part in drafting, revising a/o critically reviewing the article (30%). Rossana Cantanhede Farias de Vasconcelos: Conception, study design, execution, acquisition of data, analysis and interpretation (10%); took part in drafting, revising a/o critically reviewing the article (20%). Gustavo Limongi Moreira: Conception, study design, execution, acquisition of data, analysis and interpretation (10%); took part in drafting, revising a/o critically reviewing the article (20%). The final version to be published is in accordance with the journal to which the article has been submitted; the authors contributed equally and agreed to be accountable for all the aspects of the work.

## Disclosure

David Di Sessa, Carla de Sanctis Pecora, Rossana Cantanhede Farias de Vasconcelos, and Gustavo Limongi Moreira have been speakers for Merz Pharmaceuticals.

## Ethics Statement

This manuscript was approved by Universidade Anhanguera institution review board (CAAE: 78656124.1.0000.0347; Approval number: 6.941.771).

## Conflicts of Interest

The authors declare no conflicts of interest.

## Data Availability

The data that support the findings of this study are available from the corresponding author, upon reasonable request.
